# Current and Emerging Applications of Artificial Intelligence in Medical Imaging for Paediatric Hip Disorders—A Scoping Review

**DOI:** 10.3390/children12050645

**Published:** 2025-05-16

**Authors:** Hilde W. van Kouswijk, Hizbillah Yazid, Jan W. Schoones, M. Adhiambo Witlox, Rob G. H. H. Nelissen, Pieter Bas de Witte

**Affiliations:** 1 Department of Orthopaedics, Leiden University Medical Center, 2333 ZA Leiden, The Netherlands; 2 Department of Orthopaedic Surgery, Care and Public Health Research Institute (CAPHRI), Maastricht University, 6229 HX Maastricht, The Netherlands; 3 Department of Orthopaedics and Traumatology, Faculty of Medicine, Universitas Airlangga, Dr. Soetomo General Academic Hospital, Surabaya 60286, Indonesia; 4 Directorate of Research Policy, Leiden University Medical Center, 2333 ZA Leiden, The Netherlands

**Keywords:** artificial intelligence, hip joint, paediatric disorders, orthopaedics, medical imaging, scoping review

## Abstract

Introduction: Paediatric hip disorders present unique challenges for artificial intelligence (AI)-aided assessments of medical imaging due to disease-related and age-dependent changes in hip morphology. This scoping review aimed to describe current and emerging applications of AI in medical imaging for paediatric hip disorders. Methods: A descriptive synthesis of articles identified through PubMed, Embase, Cochrane Library, Web of Science, Emcare, and Academic Search Premier databases was performed including articles published up until June 2024. Original research articles’ titles and abstracts were screened, followed by full-text screening. Two reviewers independently conducted article screening and data extraction (i.e., data on the article and the model and its performance). Results: Out of 871 unique articles, 40 were included. The first article was dated from 2017, with annual publication rates increasing thereafter. Research contributions were primarily from China (17 [43%]) and Canada (10 [25%]). Articles mainly focused on developing novel AI models (19 [47.5%]), applied to ultrasound images or radiographs of developmental dysplasia of the hip (DDH; 37 [93%]). The three remaining articles addressed Legg–Calvé–Perthes disease, neuromuscular hip dysplasia in cerebral palsy, or hip arthritis/osteomyelitis. External validation was performed in eight articles (20%). Models were mainly applied to the diagnosis/grading of the disorder (22 [55%]), or on screening/detection (17 [42.5%]). AI models were 17 to 124 times faster (median 30) in performing a specific task than experienced human assessors, with an accuracy of 86–100%. Conclusions: Research interest in AI applied to medical imaging of paediatric hip disorders has expanded significantly since 2017, though the scope remains restricted to developing novel models for DDH imaging. Future studies should focus on (1) the external validation of existing models, (2) implementation into clinical practice, addressing the current lack of implementation efforts, and (3) paediatric hip disorders other than DDH.

## 1. Introduction

The introduction of artificial intelligence (AI) has transformed various domains in healthcare, particularly medical imaging. Paediatric hip disorders, such as developmental dysplasia of the hip (DDH), Legg–Calvé–Perthes disease (LCPD), and slipped capital femoral epiphysis (SCFE), present unique challenges for medical imaging due to the complex and age-dependent hip morphology, as well as anatomical changes due to these paediatric hip disorders. Accurate and early diagnosis of these conditions is critical to optimise treatment and prevent long-term disability [1,2,3]. Visual evaluation of imaging by radiologists, especially for larger volumes (e.g., DDH screening), can be time-consuming and generally coincides with significant intra- and interobserver variability [4]. Recently, AI applications, including machine learning and deep learning algorithms, have shown promise in enhancing the accuracy, efficiency, and reproducibility of medical imaging assessments in paediatric populations [5,6].

Despite growing interest, the integration of AI in paediatric orthopaedics remains limited, with a lack of comprehensive understanding regarding current practices, emerging trends, and critical gaps related to clinical applicability, potential benefits, and implementation challenges [7,8].

Previous studies have primarily concentrated on the use of AI in general orthopaedic imaging or in paediatric radiology—addressing areas such as bone health indices, fracture assessment, and spinal alignment—without specifically examining its role in paediatric orthopaedics [6,8,9]. Notably, paediatric hip disorders are among the most common orthopaedic conditions in children, with DDH being the most prevalent and widely screened condition in early childhood [10]. A delayed or inaccurate diagnosis and treatment of these conditions can result in severe, long-term complications [11]. This underscores the need for a dedicated review of current and emerging applications of AI in medical imaging in the full spectrum of paediatric hip disorders.

This study aimed to map the existing literature and provide a comprehensive overview of applications of AI in medical imaging for paediatric hip disorders, conducted through a scoping review.

## 2. Materials and Methods

This scoping review’s a priori protocol was registered in the Open Science Framework (OSF) database on 31 July 2024 [12]. All reporting followed the Preferred Reporting Items for Systematic reviews and Meta-Analysis extension for Scoping Reviews (PRISMA-ScR) guidelines [13].

This review followed the methodological framework originally described by Arksey and O’Malley [14] and further developed by Levac et al. [15]. The framework provides five steps to perform a descriptive synthesis of articles, which we followed for the current study: (1) identifying the research question; (2) identifying relevant articles; (3) article selection; (4) charting the data; and (5) collating, summarising, and reporting the results.

### 2.1. Stage 1: Identifying the Research Question

A Population Concept Context (PCC) framework [16] was compiled to define the topic of interest:

- Population: paediatric patients (≤18 years old) with hip disorders.
- Concept: the use of AI in medical imaging (all modalities).
- Context: applied during one or more phases of diagnostics and treatment (screening/detection, diagnostics/grading, treatment, and follow-up).

Accordingly, the following research question was formulated:

“What are the current and emerging applications of AI in medical imaging for paediatric hip disorders?”

### 2.2. Stage 2: Identifying Relevant Articles

A broad search strategy was developed based on the consensus of all authors (consisting of researchers, clinicians, and a scientific librarian). The search query consisted of a combination of keyword variations related to “Artificial intelligence”, “Medical imaging”, “Hip disorders/Orthopaedics”, and “Paediatrics”. The strategy was optimised for all consulted databases (PubMed [Supplementary File S1], Embase, Cochrane Library, Web of Science, Emcare, and Academic Search Premier), and the final search was performed on June 11, 2024. Specific in- and exclusion criteria are described in Table 1 and consisted of an extension of the PCC framework (Table 1).

### 2.3. Stage 3: Article Selection

Identified references were uploaded and de-duplicated in the web-based Rayyan screening tool (manufacturer QCRI, Doha, Qatar) [17]. Two researchers (HWvK and HY) independently screened references in two stages: (1) title and abstract (TIAB) and (2) full-text screening. Disagreements after independent TIAB or full-text screening were discussed in a consensus meeting between the two reviewers. If disagreement persisted, a third party (PBdW) was consulted.

### 2.4. Stage 4: Charting the Data

A data charting form was created a priori by two reviewers (HWvK and HY) in SPSS (version 29) [18]. During the iterative process of data charting, the form was refined to a final version. Extracted data included information on the article (e.g., first author, year of publication, and country of origin based on the primary affiliation of the first author), the research context (e.g., type of institution and objective/purpose [multiple answers possible]), the AI model (e.g., model type, its purpose, application, imaging modality, dataset sizes, and ground truth), and its performance (e.g., accuracy, sensitivity, specificity, and speed), if available. Task duration (in seconds) was recorded for both the human assessor and the model. Relative speed was then calculated as the ratio of human time to model time.

HWvK and HY each extracted the data from half of the included articles and then verified the other half (extracted by the other reviewer). A consensus meeting determined the final data extraction, after which a third party (PBdW) would be consulted if disagreement persisted.

### 2.5. Stage 5: Collating, Summarising, and Reporting the Results

The methods employed in this scoping review resulted in the collation and consolidation of existing knowledge on this topic. The review consists of the following:

1. A descriptive analysis of the included articles and mapping of the data, showing distributions of articles by time period of publication, country of origin, and study methods.
2. A narrative summary outlining the applications of the identified AI models, their central themes and focus, and their performance (presented per model application type).

## 3. Results

### 3.1. Articles on AI Models for Paediatric Hip Disorders

**Figure 1:**
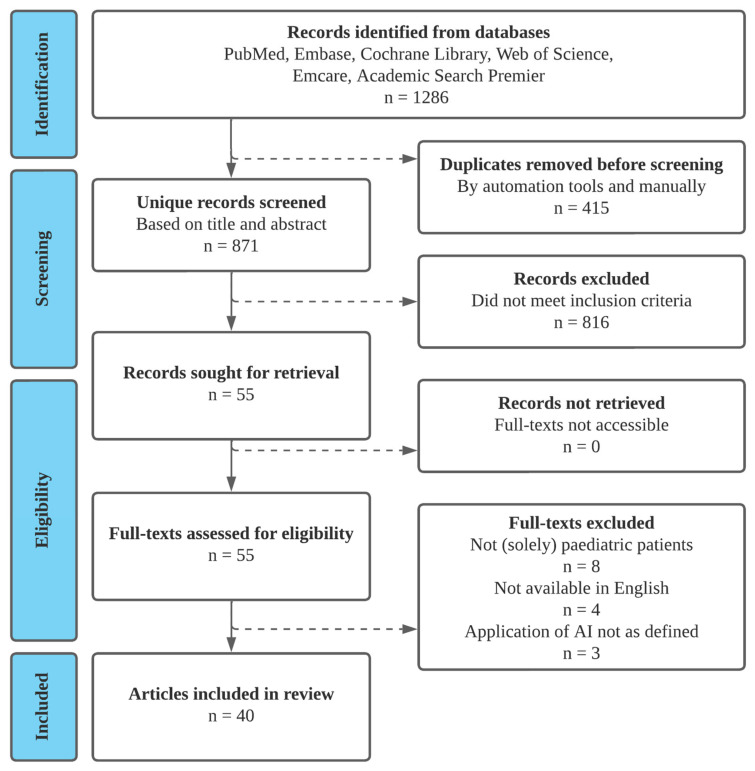
PRISMA flowchart of the inclusion process.

The systematic literature search yielded 1286 references, of which 871 were unique. TIAB and full-text screening were performed with 99.5% and 93.7% initial agreement between reviewers, respectively. In each phase of screening and extracting, agreement between reviewers was reached without the consultation of a third party. After TIAB and full-text screening, 40 articles were eligible for inclusion and underwent data extraction (Figure 1). Details of the articles excluded during full-text screening are available in Supplementary File S2; those included are listed in Supplementary File S3.

**Figure 2:**
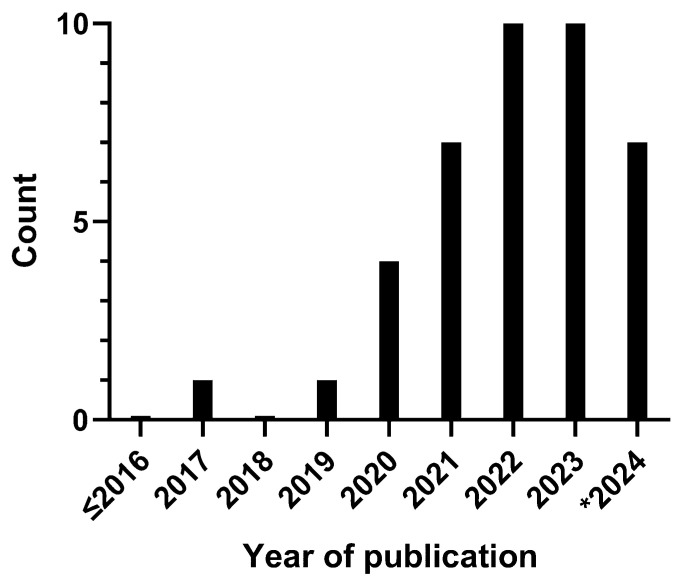
Number of publications per year (* up until June 2024).

The oldest article identified dates from 2017 [19]. Since then, the number of articles increased linearly, to 10 in 2023 and 7 in the first half of 2024 (Figure 2). A large portion of the articles had a first author affiliated in China (17 [42.5%]) [20,21,22,23,24,25,26,27,28,29,30,31,32,33,34,35,36] or Canada (10 [25%]) [19,37,38,39,40,41,42,43,44,45] (Table 2). Most investigations were performed in an academic (children’s) hospital (28 [70%]) [19,20,21,22,24,25,26,27,30,31,32,35,38,39,40,41,42,43,44,45,46,47,48,49,50,51,52,53]. The main purpose of these studies was developing a new AI model (23 [57.5%]) [19,20,21,23,24,25,26,29,30,32,33,34,35,36,40,47,48,49,50,52,54,55,56], enhancing an existing model (10 [25%]) [22,27,28,31,37,45,51,53,57,58], performing external validation (8 [20%]) [19,32,36,38,39,41,42,46], or testing a model’s feasibility for use in clinical practice (2 [5%]) [43,44].

The range in size of the datasets was 107 [41] to 303,306 [57] images (median 1321). Dataset sizes differed per purpose: new models were developed using a median of 1449 images (range 207–10,219), model enhancement was performed using a median of 330 images (range 122–303,306), external validation on a median of 675 images (range 107–2492), and feasibility was checked using either 369 [44] or 27,229 [43] images.

### 3.2. Descriptives of the AI Models

For the majority of models, the disease of interest was DDH (37 [92.5%]) [19,20,21,22,23,24,25,26,27,28,29,31,32,33,34,35,36,37,38,39,40,41,42,44,46,47,48,49,50,51,52,54,55,56,57,58] (Table 3). The remaining articles focused on either LCPD (1 [2.5%]) [53], neuromuscular hip dysplasia in cerebral palsy (CP) (1 [2.5%]) [45], or hip arthritis/osteomyelitis (1 [2.5%]) [30]. Imaging modalities to which the models were applied were ultrasound (24 [60%]) [19,20,21,23,24,25,26,28,34,37,38,39,40,41,42,43,44,46,47,49,52,54,56,57], radiographs (14 [35%]) [22,27,29,31,32,33,35,36,45,48,50,51,55,58], or magnetic resonance imaging (MRI; 2 [5%]) [30,53]. The AI models were used in clinical practice either during screening (17 [42.5%]) [20,21,24,26,28,31,32,34,37,40,41,42,47,50,56,57,58], diagnosis (22 [55%]) [19,22,23,25,27,29,33,35,36,38,39,43,44,45,46,48,49,51,52,53,54,55], or surgical planning (1 [2.5%]) [30] (Table 3). The models used were YOLO (You Only Look Once; original and enhanced versions through transfer learning) [20,22,31,55,58], MEDO Hip [38,39,41,43,44], FR-DDH [33,36], DDHnet [25], and other general convolutional neural network models (CNNs) used for transfer learning [23,26,27,28,29,30,32,33,34,35,37,45,46,47,48,50,51,52,53,54,56,57].

The tasks that the models performed were assessing image quality (e.g., whether Graf’s standard plane was present on ultrasound images or whether radiographs were tilted or rotated; 19 [47.5%]) [19,20,24,26,27,28,31,32,34,37,38,40,41,42,48,54,55,56,57], triage/screening (i.e., whether further diagnostics are necessary, dichotomous outcome; 8 [20%]) [21,23,25,30,47,50,53,58], diagnosing/grading (e.g., none/moderate/severe or Graf classification; 8 [20%]) [22,29,39,43,44,46,49,52], or performing measurements on the image (e.g., alpha or beta angles on hip ultrasound or acetabular and/or migration indexes on medical imaging; 5 [12.5%]) [33,35,36,45,51].

### 3.3. AI Model Performance

#### 3.3.1. Screening/Detection

AI models screening for DDH (17 [42.5%]) had accuracies ranging from 89% to 100% (median 96.2%) [32,34,40,41,42,47,58], sensitivity from 94% to 100% (median 98.8%), and specificity from 66% to 100% (median 97.1%) [32,40,42,47,50,58]. Comparisons between AI and experienced human assessors for DDH screening showed intraclass correlation coefficients (ICCs) ranging from 0.85 to 0.98 (median 0.93) [20,21,40,41,42,59].

#### 3.3.2. Diagnostics/Grading

AI models for DDH diagnosis/grading (20 [50%]) had an accuracy of 86% to 99% (median 94.6%) [22,23,25,35,36,46,48,52]. Sensitivity ranged from 87% to 100% (median 92.0%) and specificity from 85% to 100% (median 94.3%) [23,25,35,36,39,48,52]. ICCs between the classifications of the AI model and an experienced human assessor ranged between 0.76 and 1.00 (median 0.93) [25,35,38,49,59].

One article reported on dysplasia diagnostics in CP patients using migration percentages, reporting a sensitivity of 87.8% and a specificity of 93.4% [45]. The model’s ICC was 0.91.

For the model diagnosing LCPD, no model diagnostics were described, except for a segmentation accuracy (89.7%) [53].

#### 3.3.3. Treatment

The model identifying surgical target areas for osteomyelitis (label 1) and abscesses (label 2) around the hip joint reported an accuracy of 97.6%, sensitivity of 99.5%, and specificity of 96.9% for label 1, and 95.7%, 96.9%, and 91.5% for label 2, respectively [30].

#### 3.3.4. Speed

Seven articles (17.5%) described the time saved when using an AI model compared to experienced human assessors [25,30,32,34,35,36,45]. A diversity of radiological assessment tasks performed by experienced assessors ranged from 1 to 150 s and by AI models from 0.06 to 5 s, respectively. These tasks were performed 17 to 124 times faster (median 30) by AI models compared to experienced human assessors.

## 4. Discussion

This scoping review aimed to systematically explore and map the current applications of AI in medical imaging for paediatric hip disorders. We identified an increasing number of articles on the topic since 2017, predominantly focused on the development of new AI models applicable to DDH diagnostics. These paediatric hip disorder screening and diagnostic AI models had an accuracy ranging from 86% to 100%. Even more, the models performed up to 124 times faster than the experienced human assessor.

The specific interest in DDH for developing AI models is twofold. First, with an incidence of 1–3%, DDH is the most common paediatric hip disorder [10]. Early treatment is important in the prevention of a life-long (hip) disability [1]. Consequently, many countries perform universal or selective ultrasound screening for DDH [60], creating large image datasets for the disorder. The availability of such datasets presents opportunities for the development of AI models, as AI thrives on large volumes of data. This line of reasoning is confirmed by the articles we included, which used up to 303,000 images per study.

Secondly, the global use of hip sonography (the Graf method) for diagnosing DDH and classifying its severity underscores the value of AI algorithms in improving the sensitivity and specificity of infant hip evaluations (up to 100% in the identified models). Enhancing diagnostics is crucial, as it directly impacts treatment decisions [60]. The Graf method relies on landmarks and angles to diagnose DDH from ultrasound images [61], making the application of supervised AI models straightforward. Other high-impact paediatric hip disorders (e.g., LCPD, SCFE, and neuromuscular hip disorders) are either not categorised based on severity using a standardised method or are less common than DDH, resulting in a scarcity of data and hampering the development of AI models. The underrepresentation of these conditions in the current literature may limit the broader clinical applicability of AI models. To expand the clinical readiness of AI in medical imaging within the field of paediatric orthopaedics, future research should prioritise data collection and model development for these less-studied but clinically significant conditions.

With almost half of the included articles being published from Asian countries, specifically China, there appears to be a skewed distribution in countries developing AI applications for paediatric hip disorders. There may be several explanations for the rapid advancements in AI in Asia: (1) the New Generation AI Development Plan in China since 2017, aiming to make China lead the world in AI theory, technology, and applications [62]; (2) a larger population and therefore data availability (which fuels AI); and (3) the General Data Protection Regulation (GDPR) may slow down the deployment of AI in Europe. The development of models based on training data from one geographic region may introduce a representation bias to the model, affecting their generalisability to patient populations in countries outside of Asia [63]. Ideally, existing models that perform well within the population in which they are trained should undergo external validation and further development using datasets from populations in other geographic regions.

To ensure AI models are applicable beyond their developed setting, they must be validated on medical images acquired in different hospitals, by various examiners and/or using diverse imaging devices. This will prevent overfitting of the model and demonstrate its generalisability and robustness across clinical settings. To enable such validation efforts, initiatives promoting open-access datasets and (prospective) international multicentre collaborations are needed, ultimately facilitating the effective integration of AI models into routine clinical practice [64].

Our review identified a lack of translation to clinical practice, as current studies focus on developing new models (over and over) and generally fail to externally validate their models or test their feasibility for use in clinical practice. This issue is not only present within (paediatric) orthopaedics but is widespread across multiple medical disciplines, reflecting a systemic lack of clinical readiness of AI models [65,66,67]. In the end, models are developed to improve efficiency and/or quality of care. We therefore urge researchers to focus on the improvement and implementation of existing models rather than reinventing the AI wheel.

## 5. Strengths and Limitations

This review is the first to provide insight into the application of AI in medical imaging of paediatric hip disorders. Through our rigorous methods of independent article selection and data extraction, we ensured a comprehensive and unbiased assessment of the existing literature. It is important to note that our search strategy and the terms included, while extensive and validated by an experienced scientific librarian, were not exhaustive. Despite our best efforts, relevant publications might have been missed. As the final search was performed halfway 2024, it is likely that new studies on AI in medical imaging of paediatric hip disorders have been published by now. However, we expect potential new research to be similar to that included in our review. Our scoping review has identified several research gaps, providing directions for future research within this rapidly emerging field.

## 6. Conclusions

The application of AI in medical imaging for paediatric hip conditions is rapidly evolving, yet current efforts remain narrowly focused on DDH. Despite promising model performance, most models have not progressed beyond development. Translation to clinical practice is hindered by a lack of external validation and the underrepresentation of other hip disorders. To advance the field and realise the clinical potential of AI, future research must prioritise external validation using prospective studies, implementation into clinical practice, and the development of models for paediatric (hip) disorders other than DDH. Without this shift of focus, AI will remain a promising but unrealised tool in paediatric orthopaedic care.

## Figures and Tables

**Table 1:** In- and exclusion criteria for the current scoping review.

Inclusion Criteria	Exclusion Criteria
Quantitative studies Available in English Use of AI in medical imaging in paediatric patients (≤18 years old) with hip disorders	Qualitative studies Grey literature (e.g., theses and editorials) Conference abstracts Reviews (narrative, scoping, and systematic) Including adults (>18 years old) Imaging of joints other than the hip

**Table 2:** Descriptives of included articles (*n* = 40).

	Value
Country of origin (*n*, %) ^a^	
China	17 (42.5)
Canada	10 (25)
France	2 (5)
Japan	2 (5)
Korea	2 (5)
Turkey	2 (5)
Jordan	1 (2.5)
Mongolia	1 (2.5)
Saudi Arabia	1 (2.5)
Taiwan	1 (2.5)
United States of America	1 (2.5)
Institution type (*n*, %)	
Academic (children’s) hospital(s)	28 (70)
Peripheral (children’s) hospital(s)	12 (30)
Research purpose (*n*, %) ^b^	
Develop a new model	23 (57.5)
Enhance an existing model	10 (25)
External validation	8 (20)
Feasibility study	2 (5)
No. of images in dataset (median, range)	1321 (107–303,306)

^a^ Based on the affiliation of the article’s first author. ^b^ Articles could have multiple purposes, resulting in a total >100%.

**Table 3:** Number of studies that investigate an AI model applied to imaging of the hip stratified for imaging modality, moment of application, and indication.

	Moment of Application			Total (*n*, %)
	Screening	Diagnosis	Treatment	
Ultrasound	DDH: 13	DDH: 11	-	24 (60)
Radiographs	DDH: 4	DDH: 9 CP: 1	-	14 (35)
MRI	-	LCPD: 1	Arthritis: 1	2 (5)
Total (*n*, %)	17 (42.5)	22 (55)	1 (2.5)	40 (100)

Each study was assigned to a single category based on its primary focus. CP: cerebral palsy; DDH: developmental dysplasia of the hip; LCPD: Legg–Calvé–Perthes disease; MRI: magnetic resonance imaging.

## Data Availability

No new data were created or analysed in this study. Therefore, data sharing does not apply to this article.

## Supplementary Materials

The following supporting information can be downloaded at: https://www.mdpi.com/article/10.3390/children12050645/s1, Supplementary File S1: PubMed Search strategy, Supplementary File S2: Exclusions during full-text screening, Supplementary File S3: Included articles.
