# Supplementary material for: Current and Emerging Applications of Artificial Intelligence in Medical Imaging for Paediatric Hip Disorders—A Scoping Review
Source: Children (Basel). 2025 May 16;12(5):645. doi: 10.3390/children12050645 (PMC12110382; doi:10.3390/children12050645)
Supplement: Supplementary file 1 [file children-12-00645-s001.zip › ScR_AI_Supplementary file S1_PubMed Search strategy.pdf]

Search strategy for PubMed

("**Artificial Intelligence**"[Mesh:noexp] OR "artificial intelligence"[tw] OR "Machine Learning"[mesh] OR "Machine Learning"[tw] OR "Deep Learning"[mesh] OR "Deep Learning"[tw] OR "AI algorithm"[tw] OR "AI algorithms"[tw])

AND

("Radiography"[Mesh] OR "Radiography"[tw] OR "**radiographic imaging**"[tw] OR "Radiographic"[tw] OR "Radiogra*"[tw] OR "Diagnostic X-Ray"[tw] OR "Diagnostic X-Rays"[tw] OR "x-ray imaging"[tw] OR "x-ray image"[tw] OR "x-ray images"[tw] OR "Roentgenography"[tw] OR "Roentgenographic"[tw] OR "Roentgenogra*"[tw] OR "Rontgenography"[tw] OR "Rontgenographic"[tw] OR "Rontgenogra*"[tw] OR "X-Rays"[Mesh] OR "X-Rays"[tw] OR "X-Ray"[tw] OR "XRays"[tw] OR "XRay"[tw] OR "Ultrasonography"[Mesh] OR "Ultrasonography"[tw] OR "Ultrasonographic"[tw] OR "Ultrasonogra*"[tw] OR "echography"'[tw] OR "echographic"[tw] OR "echogra*"[tw] OR "ultrasound"[tw] OR "sonography"'[tw] OR "sonographic"[tw] OR "sonogra*"[tw] OR "Radiology"[mesh] OR "radiology"[tw] OR "radiologic"[tw] OR "radiolog*"[tw] OR "Diagnostic Imaging"[Mesh:NoExp] OR "Diagnostic Imaging"[tw] OR "Diagnostic Image"[tw] OR "Diagnostic Images"[tw] OR "medical imaging"[tw] OR "medical image"[tw] OR "medical images"[tw] OR "Image Interpretation, Computer-Assisted"[mesh] OR "Multimodal Imaging"[mesh] OR "Radiomics"[mesh] OR "Radionuclide Imaging"[mesh] OR "Tomography"[mesh] OR "Whole Body Imaging"[mesh] OR "Cardiac-Gated Single-Photon Emission Computer-Assisted Tomogra*"[tw] OR "Cardiac-Gated Single-Photon Emission Computer-Assisted Tomography"[tw] OR "Computed Tomogra*"[tw] OR "Computed Tomography "[tw] OR "Computed Tomography Angiogra*"[tw] OR "Computed Tomography Angiography"[tw] OR "Computer Assisted Tomogra*"[tw] OR "Computer Assisted Tomography "[tw] OR "Computer Tomogra*"[tw] OR "Computer Tomography "[tw] OR "Computer-Assisted Image Interpretation"[tw] OR "Computer-Assisted Radiographic Image Interpretation"[tw] OR "Dopaminergic Imag*"[tw] OR "Dopaminergic Imaging"[tw] OR "Emission-Computed Tomogra*"[tw] OR "Emission-Computed Tomography"[tw] OR "Lymphoscintigra*"[tw] OR "Lymphoscintigraphy"[tw] OR "Magnetic Resonance Imag*"[tw] OR "Magnetic Resonance Imaging"[tw] OR "MR Image"[tw] OR "MR Images"[tw] OR "MR Imaging"[tw] OR "MRI"[tw] OR "Multimodal Imaging"[tw] OR "Perfusion Imag*"[tw] OR "Perfusion Imaging"[tw] OR "PET"[tw] OR "PET"[tw] OR "Positron Emission Tomography Computed Tomogra*"[tw] OR "Positron Emission Tomography Computed Tomography"[tw] OR "Positron-Emission Tomogra*"[tw] OR "Positron-Emission Tomography"[tw] OR "Radioimmunodetect*"[tw] OR "Radioimmunodetection"[tw] OR "Radiomic*"[tw] OR "Radiomics"[tw] OR "Radionuclide Angiogra*"[tw] OR "Radionuclide Angiography"[tw] OR "Radionuclide Imag*"[tw] OR "Radionuclide Imaging"[tw] OR "Scintigra*"[tw] OR "Scintigraphy"[tw] OR "Single Photon Emission Computed Tomography Computed Tomogra*"[tw] OR "Single Photon Emission Computed Tomography Computed Tomography"[tw] OR "Single-Photon Emission-Computed Tomogra*"[tw] OR "Single-Photon

Emission-Computed Tomography"[tw] OR "SPECT "[tw] OR "SPECT "[tw] OR "Ventilation-Perfusion Scan"[tw] OR "Whole Body Imag*"[tw] OR "Whole Body Imaging"[tw] OR "X-Ray Tomograp*"[tw] OR "X-Ray Tomography"[tw])

AND

("**Hip** Dislocation"[mesh] OR "Hip Dislocation"[tw] OR "Developmental Dysplasia of the Hip"[mesh] OR "Hip Dysplasia"[tw] OR "Hip Fractures"[Mesh] OR "Hip Fractures"[tw] OR "Hip Fracture"[tw] OR "Intertrochanteric Fracture"[tw] OR "Intertrochanteric Fractures"[tw] OR "Subtrochanteric Fracture"[tw] OR "Subtrochanteric Fractures"[tw] OR "Trochanteric Fracture"[tw] OR "Trochanteric Fractures"[tw] OR "Hip Injuries"[Mesh] OR "Hip Injuries"[tw] OR "Hip Injury"[tw] OR "Osteoarthritis, Hip"[Mesh] OR "Hip Osteoarthritis"[tw] OR "Hip Prosthesis"[Mesh] OR "Hip Prosthesis"[tw] OR "Femoral Head Prosthesis"[tw] OR "Hip Contracture"[Mesh] OR "Hip Contracture"[tw] OR "Legg-Calve-Perthes Disease"[Mesh] OR "Perthes Disease"[tw] OR "Osteonecrosis of the Hip"[tw] OR "Hip Osteonecrosis"[tw] OR "Avascular Necrosis of the Hip"[tw] OR "Hip Avascular Necrosis"[tw] OR "Hip AVN"[tw] OR "SCFE"[tw] OR "Slipped Capital Femoral Epiphyses"[Mesh] OR "Slipped Capital Femoral Epiphysis"[tw] OR "SUFE"[tw] OR "Slipped Upper Femoral Epiphysis"[tw] OR "Coxa Vara"[Mesh] OR "Coxa Vara"[tw] OR "Coxa Valga"[Mesh] OR "Coxa Valga"[tw] OR "Coxa Magna"[Mesh] OR "Coxa Magna"[tw] OR "Hip Dislocation, Congenital"[Mesh] OR "Congenital Dislocation of the Hip"[tw] OR "Hip Congenital Dislocation"[tw] OR "Hip luxation"[tw] OR "Luxation of the hip"[tw] OR "Hip Subluxation"[tw] OR "Subluxation of the hip"[tw] OR "Hip"[Mesh] OR "Hip"[tw] OR "Hips"[tw] OR "Coxa"[tw] OR "Coxas"[tw] OR "Hip Joint"[Mesh] OR "Hip Joint"[tw] OR "Hip Joints"[tw] OR "Acetabulofemoral Joint"[tw] OR "Round Ligament of Femur"[tw] OR "Orthopedics"[Mesh] OR "Orthopedics"[tw] OR "Orthopedic"[tw] OR "Orthopaedics"[tw] OR "Orthopaedic"[tw] OR "Orthopedic Procedures"[Mesh] OR "Acetabuloplast*"[tw] OR "Acetabuloplasty"[tw] OR "Alveolar Bone Graft*"[tw] OR "Alveolar Bone Grafting"[tw] OR "Anterior Cruciate Ligament Reconstruction"[tw] OR "Arthrodesis"[tw] OR "Arthroplast*"[tw] OR "Arthroplasty"[tw] OR "Arthroscop*"[tw] OR "Arthroscopy"[tw] OR "Bone Lengthening"[tw] OR "Bone Transplant*"[tw] OR "Bone Transplantation"[tw] OR "Bone-Patellar Tendon-Bone Graft*"[tw] OR "Bone-Patellar Tendon-Bone Grafting"[tw] OR "Cementoplast*"[tw] OR "Cementoplasty"[tw] OR "Disarticulation"[tw] OR "Diskectom*"[tw] OR "Diskectomy"[tw] OR "Distraction Osteogenesis"[tw] OR "Fracture Fixation"[tw] OR "Genioplast*"[tw] OR "Genioplasty"[tw] OR "Hemiarthroplast*"[tw] OR "Hemiarthroplasty"[tw] OR "Hemipelvectom*"[tw] OR "Hemipelvectomy"[tw] OR "Ilizarov Technique"[tw] OR "Joint Capsule Release"[tw] OR "Kyphoplast*"[tw] OR "Kyphoplasty"[tw] OR "Laminectom*"[tw] OR "Laminectomy"[tw] OR "Limb Salvage"[tw] OR "Mandibular Osteotom*"[tw] OR "Mandibular Osteotomy"[tw] OR "Mandibular Reconstruction"[tw] OR "Maxillary Osteotom*"[tw] OR "Maxillary Osteotomy"[tw] OR "Meniscectom*"[tw] OR "Meniscectomy"[tw] OR "Open Fracture Reduction"[tw] OR "Orthognathic Surgical Procedure"[tw] OR "Orthognathic Surgical Procedures"[tw] OR "Osteotom*"[tw] OR "Osteotomy"[tw] OR "Posterior Cruciate Ligament Reconstruction"[tw] OR "Replacement Arthroplast*"[tw] OR "Replacement Arthroplasty"[tw] OR "Sinus Floor Augmentation"[tw] OR "Spinal Fusion"[tw] OR "Subchondral Arthroplast*"[tw] OR "Subchondral Arthroplasty"[tw] OR "Surgical Amputation"[tw] OR "Synovectom*"[tw] OR "Synovectomy"[tw] OR "Tendon

Transfer"[tw] OR "Tenodesis"[tw] OR "Tenotom*"[tw] OR "Tenotomy"[tw] OR "Total Disc Replacement"[tw] OR "Total Disk Replacement"[tw] OR "Traction"[tw] OR "Ulnar Collateral Ligament Reconstruction"[tw] OR "Vertebroplast*"[tw] OR "Vertebroplasty"[tw] OR "Musculoskeletal System"[Mesh:noexp] OR "musculoskeletal"[tw] OR "Skeleton"[tw] OR "Bone"[tw] OR "bones"[tw] OR "joint"[tw] OR "joints"[tw] OR "Skeleton"[mesh])

AND

("**Child**"[Mesh] OR "child"[tw] OR "children"[tw] OR "Infant"[Mesh] OR "infant"[tw] OR "infants"[tw] OR "infancy"[tw] OR "newborn"[tw] OR "newborns"[tw] OR "new-born"[tw] OR "new-borns"[tw] OR "neonate"[tw] OR "neonates"[tw] OR "neonatal"[tw] OR "neo-nate"[tw] OR "neo-nates"[tw] OR "neo-natal"[tw] OR "neonatology"[tw] OR "NICU"[ti] OR "premature"[tw] OR "prematures"[tw] OR "pre-mature"[tw] OR "pre-matures"[tw] OR "preterm"[tw] OR "pre-term"[tw] OR "postnatal"[tw] OR "post-natal"[tw] OR "baby"[tw] OR "babies"[tw] OR "suckling"[tw] OR "sucklings"[tw] OR "toddler"[tw] OR "toddlers"[tw] OR "childhood"[tw] OR "schoolchild"[tw] OR "schoolchildren"[tw] OR "childcare"[tw] OR "child-care"[tw] OR "young"[ti] OR "youngster"[tw] OR "youngsters"[tw] OR "preschool"[tw] OR "pre-school"[tw] OR "kid"[tw] OR "kids"[tw] OR "boy"[tw] OR "boys"[tw] OR "girl"[tw] OR "girls"[tw] OR "Adolescent"[Mesh] OR "adolescent"[tw] OR "adolescents"[tw] OR "adolescence"[tw] OR "pre-adolescent"[tw] OR "pre-adolescents"[tw] OR "pre-adolescence"[tw] OR "schoolage"[tw] OR "schoolboy"[tw] OR "schoolboys"[tw] OR "schoolgirl"[tw] OR "schoolgirls"[tw] OR "pre-puber"[tw] OR "pre-puberty"[tw] OR "prepuber"[tw] OR "prepubers"[tw] OR "prepuberty"[tw] OR "puber"[tw] OR "puberty"[tw] OR "puberal"[tw] OR "teenager"[tw] OR "teenagers"[tw] OR "teens"[tw] OR "youth"[tw] OR "youths"[tw] OR "underaged"[tw] OR "under-aged"[tw] OR "Pediatrics"[Mesh] OR "Pediatric"[tw] OR "Pediatrics"[tw] OR "Paediatric"[tw] OR "Paediatrics"[tw] OR "PICU"[ti] OR ("child"[all fields] NOT child[au]) OR children*[all fields] OR schoolchild*[all fields] OR "infant"[all fields] OR "infants"[all fields] OR "infancy"[all fields] OR adolesc*[all fields] OR pediat*[all fields] OR paediat*[all fields] OR neonat*[all fields] OR toddler*[all fields] OR "teen"[all fields] OR "teens"[all fields] OR teenager*[all fields] OR preteen*[all fields] OR newborn*[all fields] OR postneonat*[all fields] OR postnatal*[all fields] OR "puberty"[all fields] OR preschool*[all fields] OR suckling*[all fields] OR "juvenile"[all fields] OR "new born"[all fields] OR "new borns"[all fields] OR new-born*[all fields] OR neo-nat*[all fields] OR neonat*[all fields] OR perinat*[all fields] OR underag*[all fields] OR "under age"[all fields] OR "under aged"[all fields] OR youth*[all fields] OR kinder*[all fields] OR pubescen*[all fields] OR prepubescen*[all fields] OR "prepuberty"[all fields] OR "school age"[all fields] OR "schoolage"[all fields] OR "school ages"[all fields] OR schoolage*[all fields] OR "one year old"[ti] OR "two year old"[ti] OR "three year old"[ti] OR "four year old"[ti] OR "five year old"[ti] OR "six year old"[ti] OR "seven year old"[ti] OR "eight year old"[ti] OR "nine year old"[ti] OR "ten year old"[ti] OR "eleven year old"[ti] OR "twelve year old"[ti] OR "thirteen year old"[ti] OR "fourteen year old"[ti] OR "fifteen year old"[ti] OR "sixteen year old"[ti] OR "seventeen year old"[ti] OR "eighteen year old"[ti] OR "1 year old"[ti] OR "2 year old"[ti] OR "3 year old"[ti] OR "4 year old"[ti] OR "5 year old"[ti] OR "6 year old"[ti] OR "7 year old"[ti] OR "8 year old"[ti] OR "9 year old"[ti] OR "10 year old"[ti] OR "11 year old"[ti] OR "12 year old"[ti] OR "13 year old"[ti] OR "14 year old"[ti] OR "15 year old"[ti] OR "16 year old"[ti] OR "17 year old"[ti] OR

"18 year old"[ti] OR "two years old"[ti] OR "three years old"[ti] OR "four years old"[ti] OR "five years old"[ti] OR "six years old"[ti] OR "seven years old"[ti] OR "eight years old"[ti] OR "nine years old"[ti] OR "ten years old"[ti] OR "eleven years old"[ti] OR "twelve years old"[ti] OR "thirteen years old"[ti] OR "fourteen years old"[ti] OR "fifteen years old"[ti] OR "seventeen years old"[ti] OR "eighteen years old"[ti] OR "2 years old"[ti] OR "3 years old"[ti] OR "4 years old"[ti] OR "5 years old"[ti] OR "6 years old"[ti] OR "7 years old"[ti] OR "8 years old"[ti] OR "9 years old"[ti] OR "10 years old"[ti] OR "11 years old"[ti] OR "12 years old"[ti] OR "13 years old"[ti] OR "14 years old"[ti] OR "15 years old"[ti] OR "16 years old"[ti] OR "17 years old"[ti] OR "18 years old"[ti])
