# Supplementary material for: Current and Emerging Applications of Artificial Intelligence in Medical Imaging for Paediatric Hip Disorders—A Scoping Review
Source: Children (Basel). 2025 May 16;12(5):645. doi: 10.3390/children12050645 (PMC12110382; doi:10.3390/children12050645)
Supplement: Supplementary file 1 [file children-12-00645-s001.zip › ScR_AI_Supplementary file S2_Exclusions during full-text screening.pdf]

Articles excluded during full-text screening (n=15)

Not available in English

Kim et al. (2023) - A comparative study on keypoint detection for developmental dysplasia of hip diagnosis using deep learning models in X-ray and ultrasound images (https://dx.doi.org/10.7776/ask.2023.42.5.460)

Sha et al. (2023) - Research advances on the artificial intelligence-based imaging diagnosis of pediatric DDH (https://dx.doi.org/10.3760/cma.j.cn121113-20221116-00660)

Shen et al. (2024) - The application progress of artificial intelligence technology in the diagnosis and treatment of developmental dysplasia of the hip (https://dx.doi.org/10.3760/cma.j.cn121113-20231019-00245)

Xu et al. (2023) - Artificial intelligence technology in ultrasound screening of infant developmental dysplasia of the hip (https://dx.doi.org/10.13929/j.issn.1003-3289.2023.08.024)

Sample age >18 years

Archer et al. (2022) – AI-generated hip radiological measurements are fast and adequate for reliable assessment of hip dysplasia: An external validation study (https://dx.doi.org/10.1302/2633-1462.311.Bjo-2022-0125.R1)

Bekkouch et al. (2022) - Multi-landmark environment analysis with reinforcement learning for pelvic abnormality detection and quantification (https://dx.doi.org/10.1016/j.media.2022.102417)

Chee et al. (2018) – Performance of a deep learning algorithm in detecting osteonecrosis of the femoral head on digital radiography: A comparison with assessments by radiologists (https://dx.doi.org/10.2214/AJR.18.20817)

Kunze et al. (2022) - External Validation of a Machine Learning Algorithm for Predicting Clinically Meaningful Functional Improvement After Arthroscopic Hip Preservation Surgery (https://dx.doi.org/10.1177/03635465221124275) (also not applied as defined)

Li et al. (2019) - Auxiliary diagnosis of developmental dysplasia of the hip by automated detection of Sharp's angle on standardized anteroposterior pelvic radiographs (https://dx.doi.org/10.1097/md.0000000000018500)

Li et al. (2021) - Model construction and application for automated measurement of CE angle on pelvis orthograph based on MASK-R-CNN algorithm (https://dx.doi.org/10.1088/2057-1976/abf483)

Ruckli et al. (2023) - A Deep Learning Method for Quantification of Femoral Head Necrosis Based on Routine Hip MRI for Improved Surgical Decision Making (https://dx.doi.org/10.3390/jpm13010153)

Zeng et al. (2021) - MRI-based 3D models of the hip joint enables radiation-free computer-assisted planning of periacetabular osteotomy for treatment of hip dysplasia using deep learning for automatic segmentation (https://dx.doi.org/10.1016/j.ejro.2020.100303)

AI not applied as defined for this scoping review

Bertoncelli et al. (2020) - PredictMed: A Machine Learning Model for Identifying Risk Factors of Neuromuscular Hip Dysplasia: A Multicenter Descriptive Study (https://dx.doi.org/10.1055/s-0040-1721703)

Kim et al. (2023) - Outcome Prediction Model Following Proximal Femoral Osteotomy in Legg-Calvé-Perthes Disease Using Machine Learning Algorithms (https://dx.doi.org/10.1097/bpo.0000000000002494)

Yi et al. (2019) - Automated semantic labeling of pediatric musculoskeletal radiographs using deep learning (https://dx.doi.org/10.1007/s00247-019-04408-2)
