# Supplementary material for: Current and Emerging Applications of Artificial Intelligence in Medical Imaging for Paediatric Hip Disorders—A Scoping Review
Source: Children (Basel). 2025 May 16;12(5):645. doi: 10.3390/children12050645 (PMC12110382; doi:10.3390/children12050645)
Supplement: Supplementary file 1 [file children-12-00645-s001.zip › ScR_AI_Supplementary file S3_Included articles.pdf]

Articles included in current review (n=40)

Atalar, H., K. Ureten, G. Tokdemir, T. Tolunay, M. Ciceklidag, and O. S. Atik. 2023. 'The Diagnosis of Developmental Dysplasia of the Hip From Hip Ultrasonography Images With Deep Learning Methods', *J Pediatr Orthop*, 43: e132-e37.

Chen, J., X. Fan, Z. Chen, Y. Peng, L. Liang, C. Su, Y. Chen, and J. Yao. 2024. 'Enhancing YOLO5 for the Assessment of Irregular Pelvic Radiographs with Multimodal Information', *J Imaging Inform Med*, 37: 744-55.

Chen, T., Y. Zhang, B. Wang, J. Wang, L. Cui, J. He, and L. Cong. 2022. 'Development of a Fully Automated Graf Standard Plane and Angle Evaluation Method for Infant Hip Ultrasound Scans', *Diagnostics (Basel)*, 12.

Chen, X., S. Zhang, W. Shi, D. Wu, B. Huang, H. Tao, X. He, and N. Xu. 2023. 'A deep learning model adjusting for infant gender, age, height, and weight to determine whether the individual infant suit ultrasound examination of developmental dysplasia of the hip (DDH)', *Front Pediatr*, 11: 1293320.

Chen, Y. P., T. Y. Fan, C. C. Chu, J. J. Lin, C. Y. Ji, C. F. Kuo, and H. K. Kao. 2023. 'Automatic and Human Level Graf's Type Identification for Detecting Developmental Dysplasia of the Hip', *Biomed J*: 100614.

Den, H., J. Ito, and A. Kokaze. 2023. 'Diagnostic accuracy of a deep learning model using YOLOv5 for detecting developmental dysplasia of the hip on radiography images', *Sci Rep*, 13: 6693.

El-Hariri, H., A. J. Hodgson, K. Mulpuri, and R. Garbi. 2021. 'Automatically Delineating Key Anatomy in 3-D Ultrasound Volumes for Hip Dysplasia Screening', *Ultrasound Med Biol*, 47: 2713-22.

Fraiwan, M., N. Al-Kofahi, A. Ibnian, and O. Hanatleh. 2022. 'Detection of developmental dysplasia of the hip in X-ray images using deep transfer learning', *BMC Med Inform Decis Mak*, 22: 216.

Ghasseminia, S., A. K. S. Lim, N. D. P. Concepcion, D. Kirschner, Y. M. Teo, S. Dulai, M. Mabee, S. Kernick, C. Brockley, S. Muljadi, P. Singh, A. Rakkunedeth Hareendranathan, J. Kapur, D. Zonoobi, K. Punithakumar, and J. L. Jaremko. 2022. 'Interobserver Variability of Hip Dysplasia Indices on Sweep Ultrasound for Novices, Experts, and Artificial Intelligence', *J Pediatr Orthop*, 42: e315-e23.

Ghasseminia, Siyavash, Seyed Ehsan Seyed Bolouri, Sukhdeep Dulai, Sara Kernick, Cain Brockley, Abhilash Rakkunedeth Hareendranathan, Dornoosh Zonoobi, Padma Rao, and Jacob L. Jaremko. 2022. 'Automated diagnosis of hip dysplasia from 3D ultrasound using artificial intelligence: A two-center multi-year study', *Informatics in Medicine Unlocked*, 33.

Gong, B., J. Shi, X. Han, H. Zhang, Y. Huang, L. Hu, J. Wang, J. Du, and J. Shi. 2022. 'Diagnosis of Infantile Hip Dysplasia With B-Mode Ultrasound via Two-Stage Meta-Learning Based Deep Exclusivity Regularized Machine', *IEEE J Biomed Health Inform*, 26: 334-44.

Hareendrananthan, Abhilash Rakkunedeth, Myles Mabee, Baljot S. Chahal, Sukhdeep K. Dulai, and Jacob L. Jaremko. 2022. 'Can AI Automatically Assess Scan Quality of Hip Ultrasound?', *Applied Sciences*, 12.

Hareendranathan, A. R., B. Chahal, S. Ghasseminia, D. Zonoobi, and J. L. Jaremko. 2022. 'Impact of scan quality on AI assessment of hip dysplasia ultrasound', *J Ultrasound*, 25: 145-53.

Hareendranathan, A. R., B. S. Chahal, D. Zonoobi, D. Sukhdeep, and J. L. Jaremko. 2021. 'Artificial Intelligence to Automatically Assess Scan Quality in Hip Ultrasound', *Indian J Orthop*, 55: 1535-42.

He, J., L. Cui, T. Chen, X. Lyu, J. Yu, W. Guo, D. Wang, X. Qin, Y. Zhao, and S. Zhang. 2022. 'Study on multiplanar measurements of infant hips with three-dimensional ultrasonography', *J Clin Ultrasound*, 50: 639-45.

Huang, B., B. Xia, J. Qian, X. Zhou, X. Zhou, S. Liu, A. Chang, Z. Yan, Z. Tang, N. Xu, H. Tao, X. He, W. Yu, R. Zhang, R. Huang, D. Ni, and X. Yang. 2023. 'Artificial Intelligence-Assisted Ultrasound

Diagnosis on Infant Developmental Dysplasia of the Hip Under Constrained Computational Resources', *J Ultrasound Med*, 42: 1235-48.

Huang, T., J. Shi, J. Li, J. Wang, J. Du, and J. Shi. 2024. 'Involution Transformer based U-Net for Landmark Detection in Ultrasound Images for Diagnosis of Infantile DDH', *IEEE J Biomed Health Inform*, PP.

Jan, F., A. Rahman, R. Busaleh, H. Alwarthan, S. Aljaser, S. Al-Towailib, S. Alshammari, K. R. Alhindi, A. Almogbil, D. A. Bubshait, and M. I. B. Ahmed. 2023. 'Assessing Acetabular Index Angle in Infants: A Deep Learning-Based Novel Approach', *J Imaging*, 9.

Jaremko, J. L., A. Hareendranathan, S. E. S. Bolouri, R. F. Frey, S. Dulai, and A. L. Bailey. 2023. 'AI aided workflow for hip dysplasia screening using ultrasound in primary care clinics', *Sci Rep*, 13: 9224.

Kinugasa, M., A. Inui, S. Satsuma, D. Kobayashi, R. Sakata, M. Morishita, I. Komoto, and R. Kuroda. 2023. 'Diagnosis of Developmental Dysplasia of the Hip by Ultrasound Imaging Using Deep Learning', *J Pediatr Orthop*, 43: e538-e44.

Lee, S. W., H. U. Ye, K. J. Lee, W. Y. Jang, J. H. Lee, S. M. Hwang, and Y. R. Heo. 2021. 'Accuracy of New Deep Learning Model-Based Segmentation and Key-Point Multi-Detection Method for Ultrasonographic Developmental Dysplasia of the Hip (DDH) Screening', *Diagnostics (Basel)*, 11.

Li, C., Y. Yan, H. Xu, H. Cao, J. Zhang, J. Sha, Z. Fan, and L. Huang. 2022. 'Comparison of Transfer Learning Models in Pelvic Tilt and Rotation Measurement in Pediatric Anteroposterior Pelvic Radiographs', *J Digit Imaging*, 35: 1506-13.

Li, X., R. Zhang, Z. Wang, and J. Wang. 2024. 'Semi-supervised learning in diagnosis of infant hip dysplasia towards multisource ultrasound images', *Quant Imaging Med Surg*, 14: 3707-16.

Libon, J., C. Ng, A. Bailey, A. Hareendranathan, R. Joseph, and S. Dulai. 2023. 'Remote diagnostic imaging using artificial intelligence for diagnosing hip dysplasia in infants: Results from a mixed-methods feasibility pilot study', *Paediatr Child Health*, 28: 285-90.

Liu, C., H. Xie, S. Zhang, Z. Mao, J. Sun, and Y. Zhang. 2020. 'Misshapen Pelvis Landmark Detection With Local-Global Feature Learning for Diagnosing Developmental Dysplasia of the Hip', *IEEE Trans Med Imaging*, 39: 3944-54.

Liu, Y., L. Chen, M. Fan, T. Zhang, J. Chen, X. Li, Y. Lv, P. Zheng, F. Chen, and G. Sun. 2024. 'Application of AI-assisted MRI for the identification of surgical target areas in pediatric hip and periarticular infections', *BMC Musculoskelet Disord*, 25: 428.

Lv, Jia, Junliang Che, and Xin Chen. 2024. 'CBA-YOLOv5s: A hip dysplasia detection algorithm based on YOLOv5s using angle consistency and bi-level routing attention', *Biomedical Signal Processing and Control*, 95.

Memis, A., S. Varli, and F. Bilgili. 2020. 'Semantic segmentation of the multiform proximal femur and femoral head bones with the deep convolutional neural networks in low quality MRI sections acquired in different MRI protocols', *Comput Med Imaging Graph*, 81: 101715.

Oelen, D., P. Kaiser, T. Baumann, R. Schmid, C. Buhler, B. Munkhuu, and S. Essig. 2022. 'Accuracy of Trained Physicians is Inferior to Deep Learning-Based Algorithm for Determining Angles in Ultrasound of the Newborn Hip', *Ultraschall Med*, 43: e49-e55.

Park, H. S., K. Jeon, Y. J. Cho, S. W. Kim, S. B. Lee, G. Choi, S. Lee, Y. H. Choi, J. E. Cheon, W. S. Kim, Y. J. Ryu, and J. Y. Hwang. 2021. 'Diagnostic Performance of a New Convolutional Neural Network Algorithm for Detecting Developmental Dysplasia of the Hip on Anteroposterior Radiographs', *Korean J Radiol*, 22: 612-23.

Perry, Sheridan, Matthew Folkman, Takara O'Brien, Lauren A. Wilson, Eric Coyle, Raymond W. Liu, Charles T. Price, and Victor A. Huayamave. 2024. 'Unaligned Hip Radiograph Assessment Utilizing Convolutional Neural Networks for the Assessment of Developmental Dysplasia of the Hip', *Journal of Engineering and Science in Medical Diagnostics and Therapy*, 7.

Pham, T. T., M. B. Le, L. H. Le, J. Andersen, and E. Lou. 2021. 'Assessment of hip displacement in children with cerebral palsy using machine learning approach', *Med Biol Eng Comput*, 59: 1877-87.

Quader, N., A. J. Hodgson, K. Mulpuri, E. Schaeffer, and R. Abugharbieh. 2017. 'Automatic Evaluation of Scan Adequacy and Dysplasia Metrics in 2-D Ultrasound Images of the Neonatal Hip', *Ultrasound Med Biol*, 43: 1252-62.

Sezer, A., and H. B. Sezer. 2020. 'Deep Convolutional Neural Network-Based Automatic Classification of Neonatal Hip Ultrasound Images: A Novel Data Augmentation Approach with Speckle Noise Reduction', *Ultrasound Med Biol*, 46: 735-49.

Sezer, A., and H. B. Sezer. 2023. 'Segmentation of measurable images from standard plane of Graf hip ultrasonograms based on Mask Region-Based Convolutional Neural Network', *Jt Dis Relat Surg*, 34: 590-97.

Sha, J., L. Huang, Y. Chen, J. Lin, Z. Fan, Y. Li, and Y. Yan. 2023. 'A novel approach for screening standard anteroposterior pelvic radiographs in children', *Eur J Pediatr*, 182: 4983-91.

Wu, Q., H. Ma, J. Sun, C. Liu, J. Fang, H. Xie, and S. Zhang. 2022. 'Application of deep-learning-based artificial intelligence in acetabular index measurement', *Front Pediatr*, 10: 1049575.

Xu, J., H. Xie, C. Liu, F. Yang, S. Zhang, X. Chen, and Y. Zhang. 2021. 'Hip Landmark Detection With Dependency Mining in Ultrasound Image', *IEEE Trans Med Imaging*, 40: 3762-74.

Xu, W., L. Shu, P. Gong, C. Huang, J. Xu, J. Zhao, Q. Shu, M. Zhu, G. Qi, G. Zhao, and G. Yu. 2021. 'A Deep-Learning Aided Diagnostic System in Assessing Developmental Dysplasia of the Hip on Pediatric Pelvic Radiographs', *Front Pediatr*, 9: 785480.

Zhang, S. C., J. Sun, C. B. Liu, J. H. Fang, H. T. Xie, and B. Ning. 2020. 'Clinical application of artificial intelligence-assisted diagnosis using anteroposterior pelvic radiographs in children with developmental dysplasia of the hip', *Bone Joint J*, 102-B: 1574-81.
